# Prolonged maternal investment in northern bottlenose whales alters our understanding of beaked whale reproductive life history

**DOI:** 10.1371/journal.pone.0235114

**Published:** 2020-06-23

**Authors:** Laura Joan Feyrer, Shu ting Zhao, Hal Whitehead, Cory J. D. Matthews

**Affiliations:** 1 Department of Biology, Dalhousie University, Halifax, Nova Scotia, Canada; 2 Arctic Aquatic Research Division, Fisheries and Oceans Canada, Winnipeg, Canada; Hawaii Pacific University, UNITED STATES

## Abstract

Nursing and weaning periods are poorly understood in cetaceans due to the difficulty of assessing underwater behaviour in the wild. However, the onset and completion of weaning are critical turning points for individual development and survival, with implications for a species’ life history including reproductive potential. δ^15^N and δ^13^C deposited in odontocete teeth annuli provide a lifetime record of diet, offering an opportunity to investigate variation and trends in fundamental biology. While available reproductive parameters for beaked whales have largely been inferred from single records of stranded or hunted animals and extrapolated across species, here we examine the weaning strategy and nursing duration in northern bottlenose whales (*Hyperoodon ampullatus*) by measuring stable isotopes deposited in dentine growth layer groups (GLGs). Using a collection of *H*. *ampullatus* teeth taken from whales killed during the whaling era (N = 48) and from two stranded specimens, we compared ontogenetic variation of δ^15^N and δ^13^C found in annual GLGs across all individuals, by sex and by region. We detected age-based trends in both δ^15^N and δ^13^C that are consistent across regions and males and females, and indicate that nursing is prolonged and weaning does not conclude until whales are 3–4 years old, substantially later than previous estimates of 1 year. Incorporating a prolonged period of maternal care into *H*. *ampullatus* life history significantly reduces their reproductive potential, with broad implications for models of beaked whale life history, energetics and the species’ recovery from whaling.

## Introduction

Maternal investment in mammals varies based on an array of ecological and evolutionary factors resulting in a range of maternal strategies e.g. [1]. Nursing is critical to the survival and fitness of infant mammals; providing our earliest energetic and nutritional requirements, supporting maternal bonding, and initializing ongoing socialization [2–4]. Nursing duration and the weaning strategy have implications for infant survival, interbirth interval, and lifetime reproductive output, which are critical measures for understanding the life history, energetics and population dynamics of a species [5]. While lactation may occur over a period of weeks to years, weaning initiation and completion are important developmental turning points–as juveniles become nutritionally independent, it allows females to redirect significant energetic resources back to themselves and towards their future offspring [6,7]. Weaning, which may be sudden or gradual, depends on a range of factors including the survival and vulnerability of offspring in the postpartum period, the technical difficulty of self-sufficient foraging strategies, species social structure, individual behavioural plasticity and regional prey availability [3,5,8,9]. Responding to a range of ecological and evolutionary factors, nursing duration can vary widely among and even within species, forming the context of the weaning “conflict”, with trade-offs between the fitness of offspring and future female reproductive potential [3,4,10].

Maternal investment in cetaceans (dolphins and whales) is known to be extensive and provides a key role in infant survival, however our appreciation of weaning strategies is challenged by the cryptic nature of nursing behaviour and their aquatic habitat [1]. What we do know can be generalized by sub-order; with a large degree of variability between species, odontocetes appear to prolong nursing and weaning over years (mean = 21 months), while mysticetes typically wean their young within the first year (mean = 11 months) [11] (S1 Table). This difference in maternal investment has been linked to energetic resources available to income versus capital breeders [1,12].

Four different methods have been used to estimate nursing duration in cetaceans: stomach content analysis, cow-calf ratios, behavioural observations and stable isotope analyses, which may explain some of the discrepancies between estimates within and among species [1,11]. Across studies, behavioural observations typically reported the oldest average age at weaning (27 months), in contrast with stomach content analyses, which found average weaning age occurred much younger (16 months, S1 Table). Temporal analysis of nitrogen stable isotopes (δ^15^N) in accretionary tissues, such as sequential growth layer groups (GLGs) in dentine, have also been used to estimate weaning age and other ontogenetic shifts in individual foraging and trophic level based on nutritional physiology [13–17]. As δ^15^N decreases during the transition from juveniles feeding exclusively on milk to independent foraging, differences in δ^15^N between GLGs in tooth dentine can be used to estimate nursing duration and weaning completion e.g. [14,16]. However weaning related relationships with δ^13^C are less clear and across studies there is no consistent trend or pattern reported for isotopic carbon found in marine mammal tissues during the dietary transition from milk to prey (e.g. [16–18]).

Due to the offshore habitat and elusive nature of deep diving beaked whales (Ziphiidae), there is a lack of baseline data on key aspects of their life history so that reproductive parameters are poorly understood [19]. Much of our understanding of their biology comes from one species, the northern bottlenose whale (*Hyperoodon ampullatus*), which was the target of a century of commercial whaling across the North Atlantic ending in the early 1970’s. In the final years of the commercial hunt in Labrador and northern Iceland, data otherwise difficult to collect today using non-lethal methods were recorded for many individuals, including age (from teeth), sex, sexual maturity, reproductive state, fetal term, and stomach contents [20]. Whaling records for the species provide the only estimates of reproductive parameters, which have been the basis for previous studies of beaked whale energetics (e.g. [5,21]) and include: gestation (12 months)—based on fetal growth curves; lactation length (~ 1 year)—based on a single calf that had both milk and squid in its stomach; resulting in a combined estimate of calving interval (2 years), which was also supported by an accumulation of 0.5 corpora per year in mature females [20].

Similar to other odontocetes, dentine GLGs in *H*. *ampullatus* form annually deposited layers which have been used to age individual specimens [22,23]. However beaked whales are unique among odontocetes in that most only have a single pair of tusk-like teeth that erupt in mature males and remain embedded in the jaw of juveniles and females [24]. Likely due to the difficulty in accessing tooth specimens, this study is the first investigation of ontogenetic diet shifts using stable isotopes for any species of beaked whale, based on samples from an unusually large collection (N = 151 individuals) of *H*. *ampullatus* teeth taken from whales that were commercially hunted in the North Atlantic.

Our primary objective was to characterize nursing duration and the end of the weaning period in individual *H*. *ampullatus* using δ^15^N and δ^13^C, accounting for potential differences due to sex or regional variation. We test the hypothesis that nursing extends beyond one year, in contrast to Benjaminsen & Christensen’s [20] inference based on stomach contents of a single calf. Similar to *Physeter macrocephalus*, another deep diving cetacean with prolonged maternal care [25], beaked whales regularly dive to extreme depths (~1000m) to feed on mesopelagic and epibenthic prey [19]. As a result, juveniles may not be physically capable of independent foraging until they have grown large enough to be competent divers or engage in demanding foraging strategies, the complexities of which are currently poorly understood. Secondarily we compare differences in diet between juveniles and adults to assess whether, similar to other odontocetes (e.g. *Orcinus orca* [14]; *P*. *macrocephalus* [13]), there is evidence of increases in dietary trophic level with age. This study offers a rare opportunity to expand our appreciation of the variation in maternal investment strategies in beaked whales and across cetaceans.

## Materials and methods

### Tooth collection and dentine sampling

**Fig 1:**
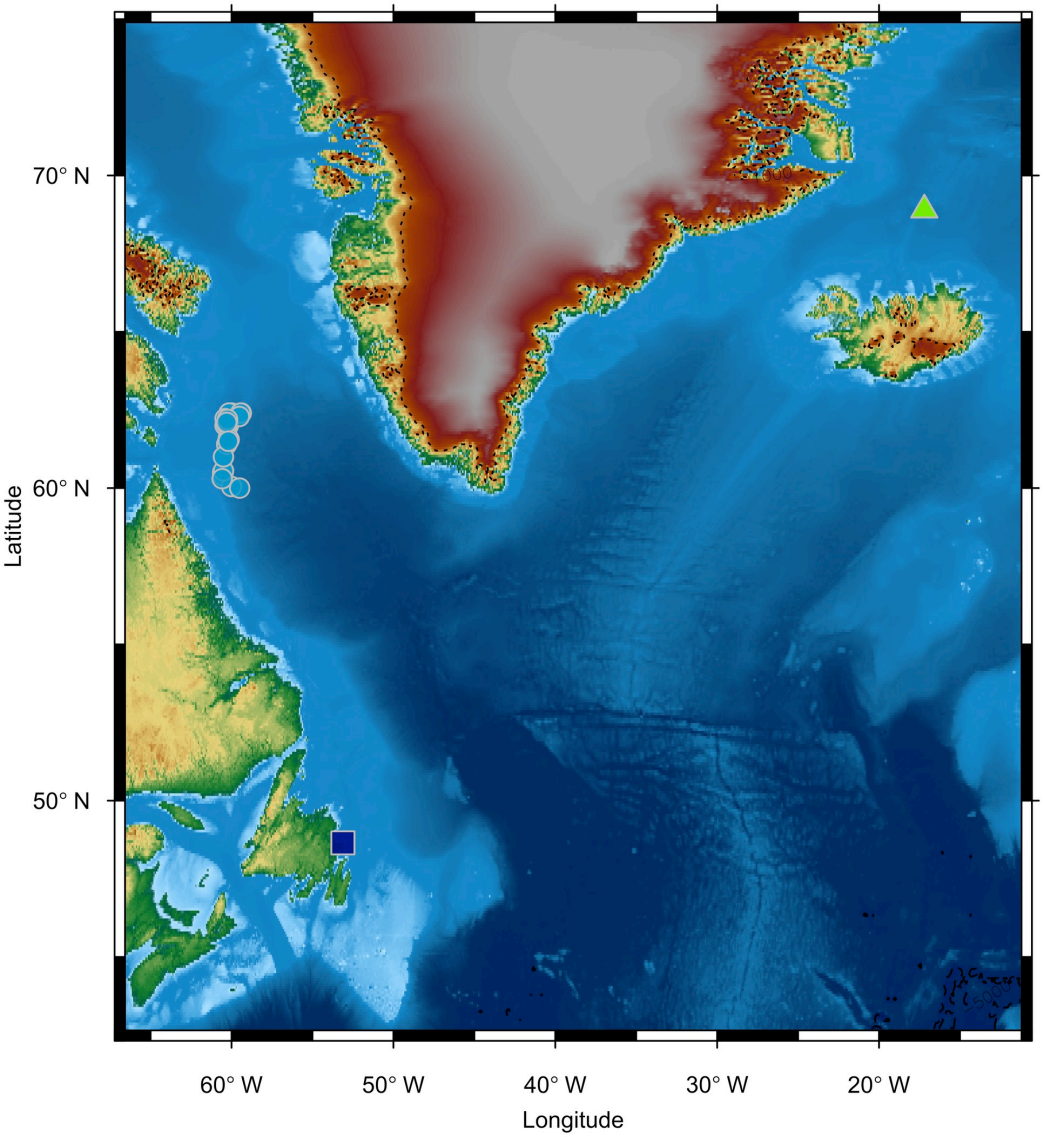
Map of study area regions and specimen collection locations. Green triangle = Iceland, light blue dots = Northern Labrador, dark blue square = Newfoundland strandings.

Teeth were taken from *H*. *ampullatus* killed by Norwegian whalers in the waters off northern Iceland in 1967 and northern Labrador in 1971 [26] (Fig 1). Northern bottlenose whales are usually found in groups of one to four, and whalers would take all the whales they encountered, regardless of sex or age class, so we assume our dataset has low demographic capture bias [20]. Individuals included in this analysis ranged from 4–27 years old (median age = 14). The teeth of two *H*. *ampullatus* that stranded in northeast Newfoundland in 2004 were also analyzed. As specimens were part of an archived natural history collection, no approval from the University Committee on Laboratory Animals was required.

**Fig 2:**
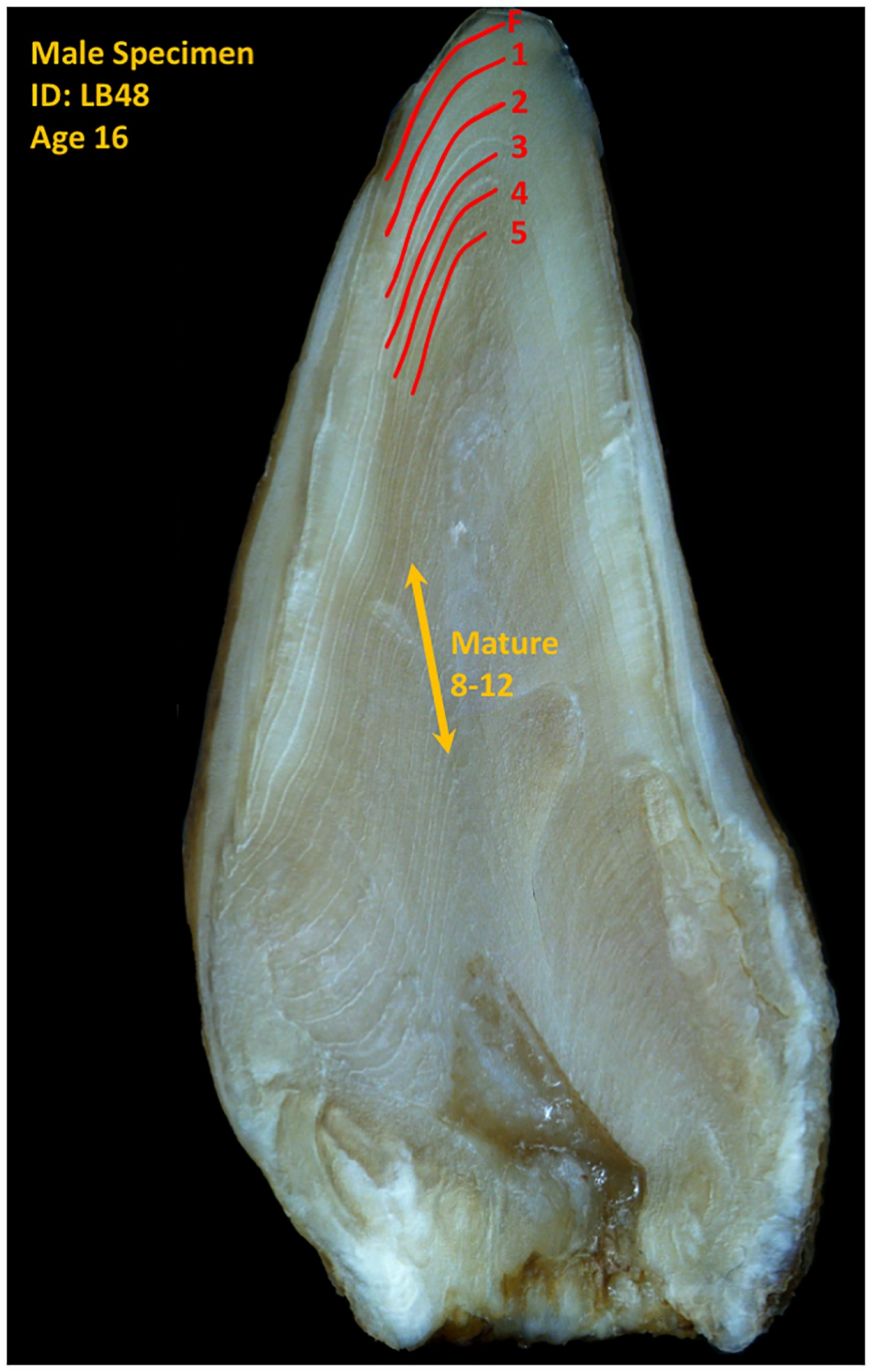
A sectioned *H*. *ampullatus* tooth prior to sampling. GLGs are annotated: F = fetal, 1–5 = years (red lines) and mature = sampling across years 8–12 (yellow line).

The jaws of whaled specimens were originally boiled for two hours to facilitate tooth extraction [26]. Teeth were sectioned along the longitudinal midline and stored unpreserved at room temperature in individual sachets for over 40 years prior to this study. Genetic analysis of gum-tissue from the teeth used in this study confirmed the sex documented in the whaling records [27,28]. The teeth from Newfoundland animals were extracted from decomposed specimens, air dried and stored whole until being sectioned for this study. Similar to other odontocetes [29–31], *H*. *ampullatus* dentine is laminated, with one clear and one opaque layer defining each annual GLG within the cone of the tooth [22] (Fig 2). Only teeth with a clear neo-natal line and defined GLG structure across the first five years were retained for isotope analysis, reducing our sample size to 50 individuals (N = 6 from Iceland, N = 42 from Labrador, N = 2 from Newfoundland). To improve GLG definition, tooth sections were initially polished using 30μm aluminum oxide lapping film [16] and then acid-etched using 10% formic acid [32]. GLGs were counted and aged assuming annual deposition, starting at the line that divides prenatal and postnatal dentine [16,22]. Using a single section of each tooth, GLGs 1–5 were sampled individually at a depth of 250-μm with a 300-μm-diameter drill bit, using a high-resolution micro-mill (New Wave Research, Freemont, California). When sufficient prenatal dentine was present it was sampled at a depth of 150 μm. For mature individuals (> 9 years old) [26], we also collected samples from older GLGs as a proxy for adult diet (N = 29). However, as whales age their GLGs become compressed and are not wide enough to sample individually. Instead we collected samples representative of the mature age class by drilling across GLGs 8–12 as a group with a 1 mm-diameter drill bit using a Dremel hand tool.

### Stable isotope analysis (δ^15^N / δ^13^C)

Powdered dentine from each sampled GLG was weighed (~1 mg) into tin cups for isotopic analysis on a Vario EL Cube elemental analyzer (Elementar, Germany) connected to a DELTA Advantage isotope ratio mass spectrometer (Thermo, Germany). Isotope ratios are reported in Delta notation (δ) as per mil (‰) deviation from isotope ratios of atmospheric N_2_ for nitrogen and Vienna Pee-Dee Belemnite (V-PDB) limestone for carbon. δ^15^N or δ^13^C are defined as δ = (R_sample_−R_standard_)/R_standard_), where R is the ratio of the abundance of the heavy to the light isotope. Values are normalized to internal standards nicotinamide, ammonium sulfate + sucrose, caffeine, and glutamic acid, whose isotopic compositions cover the natural range of samples (δ^15^N -16.61 to 16.58‰, δ^13^C -34.46 to ‒11.94‰) and are calibrated to international standards IAEA-N1(+0.4‰), IAEA-N2(+20.3‰), USGS-40(-4.52‰) and USGS-41(47.57‰) for δ^15^N, and IAEA-CH-6(-10.4‰), NBS-22(-29.91‰), USGS-40(-26.24‰) and USGS-41(37.76‰) for δ^13^C. Analytical precision based on repeated measures of laboratory reference materials not used in calibrations was ~0.1‰ for both δ^15^N and δ^13^C within multiple laboratory runs. Variation between duplicate measures of ~10% of samples had an absolute mean of 0.26 ‰ for δ^15^N and 0.21 ‰ for δ^13^C.

The small size of some GLGs meant it was sometimes necessary to collect amounts less than 1 mg. A linearity study showed samples <0.5 mg appeared to have a positive bias in δ^15^N but not δ^13^C, and further analysis was restricted to samples weighing >0.5mg, reducing the number of GLG samples available for some individuals. Additionally, we omitted the smallest duplicate sample, so that only a single sample from an individual GLG was included in further analysis [33].

### Data analysis

Following the screening for duplicates and sample weight described above, 50 individuals were included in summary statistics regardless of how many GLGs were available. However, ontogenetic trend analysis was restricted to those individuals which had stable isotope data available from at least GLGs 1–3 (N = 37). Data structure, variables, and sample sizes are identified in Table 1 and variable inclusion rationale and data sources are further described in S2 Table.

**Table 1:** Data structure, variables and sample sizes.

Dependent variables	Independent variables	Total individuals	GLG chronologies
		N = GLG samples	N = GLG samples
**δ^15^N**	**Region**	**50 IDs**	**39 IDs**
**δ^13^C**	**GLG–Year, Age Class**	**N = 244 GLGs**	**N = 207 GLGs**
	**Sex**	(*288 including duplicate samples)	
	**Individual Age**		

For comparison with other published values and ecological studies, carbon isotope values were adjusted for the oceanic Suess effect, applying a factor of 0.0019‰ yr ^-1^ to δ ^13^C measured in GLGs; δ ^13^C_cor_ values are approximately relative to the year 2000 [16,34,35]. The isotope values sampled from a cross section of mature GLGs (age 8–12) were assumed to represent the average isotopic profile of adult whales, and used as a benchmark for assessing when the weaning associated δ^15^N decline ended.

The dataset was initially summarized and explored for the presence of ontogenetic trends in nitrogen and carbon isotope ratios. The effect of sex and region on isotopic composition was initially evaluated using two-sample t-tests. A hierarchical linear mixed effects regression model implemented with the lme4 package in R (Version 3.0.1 [36]) assessed the effects of sex, region and GLG. Given uneven sample sizes between GLGs, we used a paired t-test to consider the distinction between subsequent GLGs. Due to the small sample size (N = 2) and differences in source collection from other samples, Newfoundland specimens were not included in statistical summaries or tests unless specified.

To investigate ontogenetic trends and nursing duration, for each individual with samples from GLGs 1–3 (N = 37) we calculated the ‰ difference between GLG 1 and all other available GLGs (fetal dentine, GLGs 2-maturity). Three methods of determining weaning completion were compared for individuals which had samples collected from mature age classes by calculating the age: (A) when δ^15^N values stopped decreasing (e.g. the lowest value of δ^15^N in the chronology, [18]; (B) when δ^15^N was equal to the value for their mature age class value (+/- 0.25 ‰) [14]; and (C) when δ^15^N was -1.2‰ lower than GLG1(+- 0.25 ‰) [14,16]. The threshold for (C) was based on an average ‰ difference between GLG1 and mature samples in this study, and similar differences found in other studies of weaning in odontocetes [14,16]. For each method, individual age at weaning completion was compared by sex and between Labrador and Iceland regions using a two-sample t-test. Small sample size for Newfoundland precluded inclusion in significance tests.

## Results

### Nitrogen

**Fig 3:**
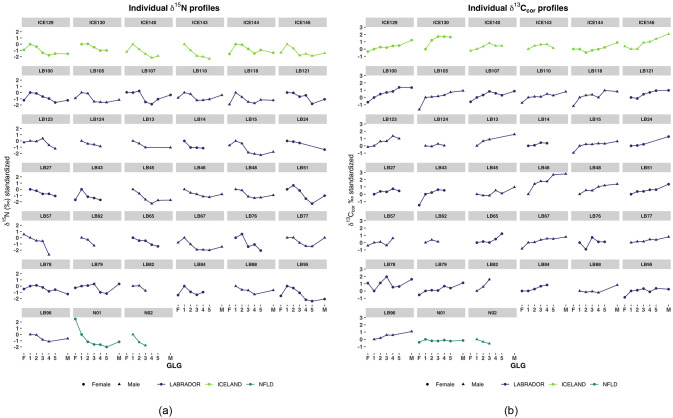
Individual chronologies for (a) δ ^15^N and (b) δ ^13^C for each region. Regions are indicated by colour. Isotope values were standardized to be relative to GLG 1 for prior (fetal dentine = F) or subsequent (years 2—mature = M) GLGs. Sex of specimen is indicated by circle (female) and triangles (male).

Across individual chronologies, we found δ^15^N generally peaked in GLG1 (mean = 17.73, SE = 0.10) and then declined with age. Within individuals, the relative decline in δ^15^N between GLG 1 and all other GLG years averaged– 1.02 ‰ (Fig 3a). GLG 1 δ^15^N was higher (mean = 0.93 ‰) than fetal dentine (mean = 17.00, SE = 0.16) and 1.06 ‰ higher than mature age class values (mean = 16.62, SE = 0.09). δ^15^N values across all GLGs from Labrador and Iceland ranged ~3.8 ‰ (15.16 to 19.0‰). For the two specimens from Newfoundland, δ^15^N spanned 4.8‰ and was lower (range 12.9–17.7‰) than average values from Labrador and Iceland. For GLGs > 1, both Newfoundland specimens were greater than 1 standard deviation lower in δ^15^N than other regions, with the adult female ~3‰ lower across GLGs.

### Carbon

δ^13^C_cor_ values generally increased with age (mean increase in δ^13^C_cor_ per GLG = 0.37, fetal to maturity). GLG1 was on average more enriched in ^13^C_cor_ (+0.51 ‰, mean = -14.07) than fetal dentine (mean = -14.48), and more depleted than older GLGs. The range of δ^13^C_cor_ values for mature samples were on average 1.06 ‰ higher than GLG 1 (Fig 3b). Between regions, δ^13^C_cor_ in Labrador and Iceland were higher (-15.66 to -12.57 ‰) than Newfoundland (-17.17 to—14.78‰). The juvenile male whale from Newfoundland was one notable exception to the overall ontogenetic increase in carbon, as his δ ^13^C_cor_ values declined from GLG 1 to 3 (Fig 3b). Across all GLGs, δ^13^C_cor_ values for the Newfoundland specimens were > 1 standard deviation below Labrador or Iceland specimen GLGs.

### Influence of sex, region and GLG

**Fig 4:**
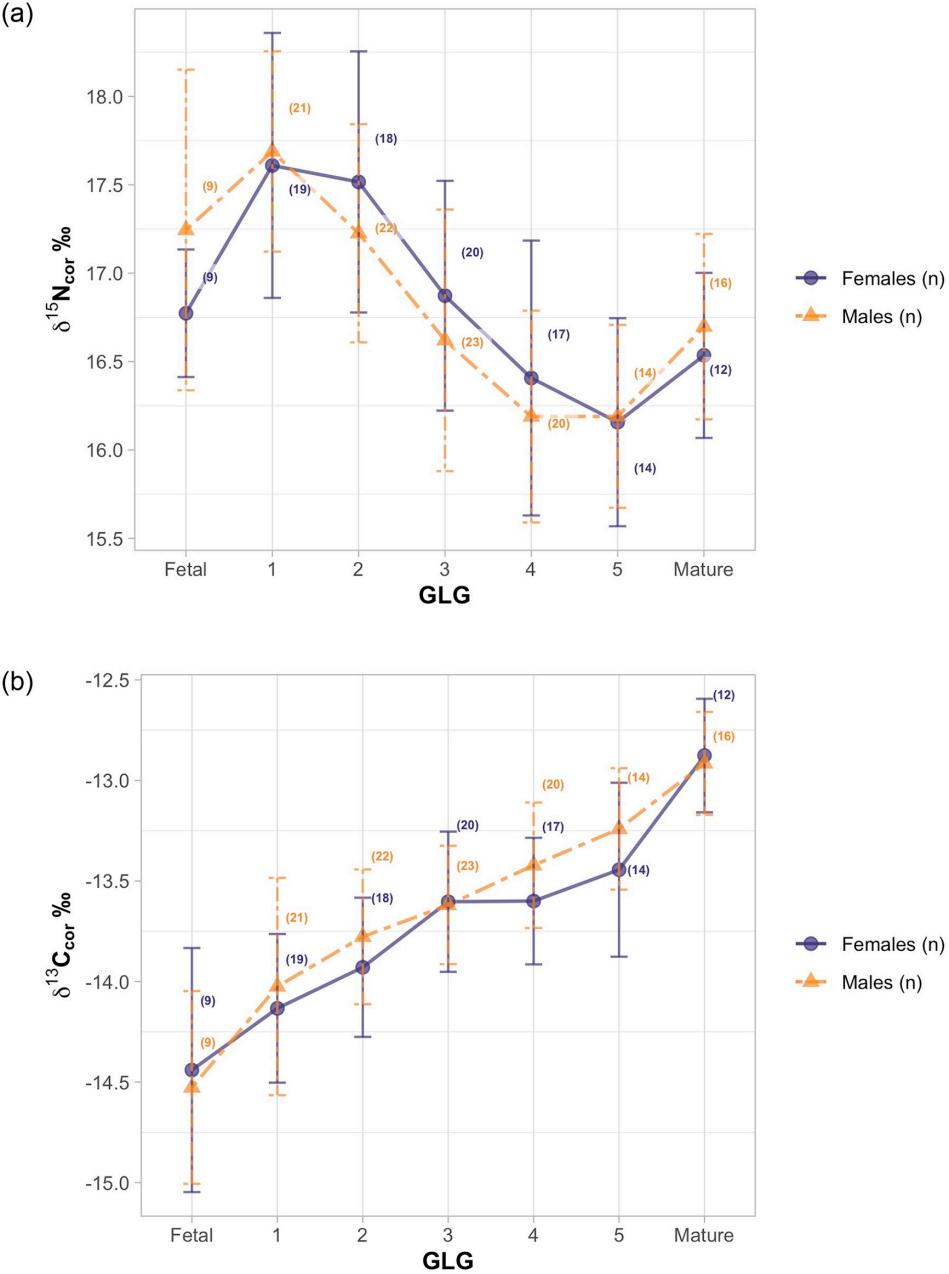
Ontogenetic trends in average (a) δ^15^N and (b) δ^13^C by sex. Females (N = 109 GLG samples) are purple points and males (N = 125 GLG samples) are orange triangles. Whisker bars represent standard deviation. Iceland and Labrador samples only.

**Fig 5:**
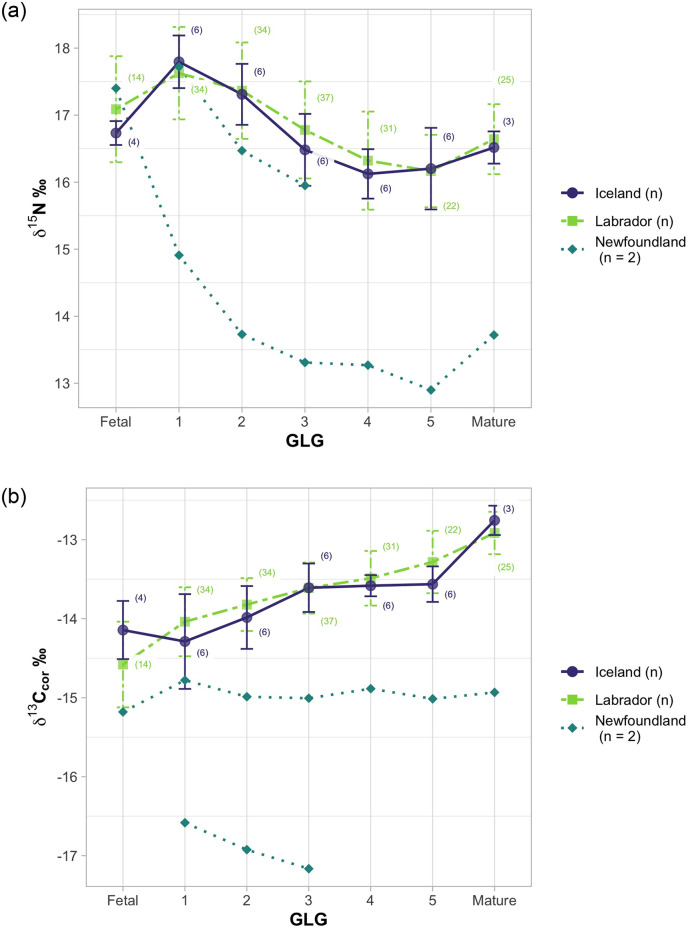
Ontogenetic trends by region for values of (a) δ^15^N and (b) δ^13^C. Purple points and green squares are mean values with standard deviation for Labrador and Iceland, blue diamonds are individual values of two specimens from Newfoundland.

Average values of δ^15^N and δ^13^C_cor_ for females and males had considerable overlap and did not demonstrate a consistent pattern or significant difference between sexes across GLG’s (Fig 4a and 4b, *t* = 0.85, df = 53.2, *p* = 0.39). Differences in values of δ^15^N and δ^13^C_cor_ between Labrador and Iceland were not significant (Fig 5a and 5b, *t* = 0.58, df = 12.1, *p* = 0.57).

Mixed effects models, implementing individual as a random effect, compared 8 different combinations of fixed effects including GLG, Region and Sex (Table 2a and 2b). Only Region and GLG were retained in the best fit mixed effect models for predicting relative δ^15^N and δ^13^C values. Model fit, assessed using Δ AIC ≦ 2, indicated GLG was important for explaining both δ^15^N and δ^13^C, Region was included in all best fit models for δ^15^N and in one model for δ^13^C, Sex was also included in one of the best models for δ^13^C (Table 2). Given the overlap in mean values with standard error between Labrador and Iceland and between males and females we conclude that the influence of region and sex on isotopic profiles are small relative to the variation attributed to GLG (age) and individual.

**Table 2:** Mixed effect model results comparisons for (a) δ^15^N and (b) δ^13^C. Best fit models are indicated in bold based on lowest AIC score and Δ AIC ≦ 2. BIC and Log Likelihood (logLik) scores with degrees of freedom (df) are included for comparison. “(1|ID)” indicates an individual effect.

**(a)**					
**δ^15^N**	**ΔAIC**	**AIC**	**BIC**	**logLik**	**df**
**~GLG_N+Region +(1|ID)**	**0.0**	**329.3**	**345.1**	**-159.6**	**5**
**~Sex +GLG_N+Region +(1|ID)**	**0.5**	**329.8**	**348.7**	**-158.9**	**6**
~GLG_N*Region +(1|ID)	**1.5**	**331.3**	**350.2**	**-159.6**	**6**
~GLG_N +(1|ID)	2.3	331.6	344.2	-161.8	4
~Sex*GLG_N+Region +(1|ID)	2.4	331.6	353.8	-158.8	7
~Sex*GLG_N+(1|ID)	2.7	334.3	353.3	-161.1	6
~Sex+GLG_N +(1|ID)	3.2	332.5	348.3	-161.2	5
~Sex+Region +(1|ID)	25.1	354.3	370.1	-172.2	5
~1 +(1|ID)	26.9	356.2	365.7	-175.1	3
**(b)**					
**δ^13^C**	**ΔAIC**	**AIC**	**BIC**	**logLik**	**df**
**~GLG_N +(1|ID)**	**0.0**	**198.9**	**211.6**	**-95.5**	**4**
**~GLG_N+Region +(1|ID)**	**1.0**	**199.9**	**215.7**	**-94.9**	**5**
**~Sex+GLG_N +(1|ID)**	**2.0**	**200.9**	**216.7**	**-95.4**	**5**
~GLG_N*Region +(1|ID)	2.2	201.1	220.0	-94.5	6
~Sex*GLG_N +(1|ID)	2.7	201.6	220.6	-94.8	6
~Sex +GLG_N +Region+(1|ID)	2.9	201.8	220.8	-94.9	6
~Sex*GLG_N +Region+(1|ID)	3.6	202.5	224.6	-94.3	7
~1 +(1|ID)	135.8	334.7	344.2	-164.3	3
~Sex + Region + (1|ID)	139.0	337.9	353.7	-164.0	5

Paired t-tests assessing the difference between δ^15^N and δ^13^C of an individual between consecutive GLG’s found significant differences between δ^15^N in GLG pairs 1 through 4 and between δ^13^C in GLG pairs Fetal (F) through age 3 (Table 3). GLGs 5 and mature (M) were also significantly different for both isotopes.

**Table 3:** Paired t-test results for comparisons between GLG years within individuals for (a) δ^15^N and (b) δ^13^C. Test significance (p-value), mean difference in ‰ (Mean dif. ‰), confidence intervals of the difference (C.I. ‰) and degrees of freedom (df) are presented for each test.

**(a)**					
**δ^15^N**					
**GLG**	**p-value**	**Mean dif. (‰)**	**C.I. (‰)**		**df**
**F to 1**	0.005	-0.72	-1.23	-0.26	18
**1 to 2**	<0.001	0.37	0.22	0.52	38
**2 to 3**	<0.001	0.65	0.50	0.81	40
**3 to 4**	<0.001	0.40	0.24	0.57	37
**4 to 5**	0.322	0.09	-0.10	0.28	28
**5 to M**	0.001	-0.49	-0.74	-0.23	20
**(b)**					
**δ^13^C**					
**GLG**	**p-value**	**Mean dif. (‰)**	**CI (‰)**		**df**
**F to 1**	0.003	-0.51	-0.81	-0.20	18
**1 to 2**	0.002	-0.22	-0.36	-0.09	38
**2 to 3**	<0.001	-0.24	-0.36	-0.12	40
**3 to 4**	0.225	-0.09	-0.23	0.06	37
**4 to 5**	0.086	-0.13	-0.28	0.02	28
**5 to M**	<0.001	-0.35	-0.53	-0.17	20

### Weaning completion

Nursing duration ranged across methods with median age of three to five. Method (A) provided older estimates of weaning completion (mean = 4.5), while methods (B) and (C) suggested weaning was completed earlier, with mean ages of 3.4. There was no substantial difference in nursing duration between Labrador or Iceland regions or with sex (Table 4) using any of the weaning analysis methods.

**Table 4:** Mean, median and range of weaning completion age for different estimation methods as described in the analyses, compared by (a) sex and (b) region.

**(a)**				
**Estimation Method**	**Sex**	**Mean GLG**	**Median GLG**	**GLG Range (yrs)**
**A**	**F**	**4.6**	**5**	**4–5**
	**M**	**4.4**	**5**	**3–5**
**B**	**F**	**3.7**	**4**	**2–5**
	**M**	**3.1**	**3**	**2–5**
**C**	**F**	**3.4**	**3**	**2–5**
	**M**	**3.1**	**3**	**2–5**
**(b)**				
**Estimation Method**	**Region**	**Mean GLG**	**Median GLG**	**GLG Range (yrs)**
**A**	**Iceland**	**4.3**	**4**	**4–5**
	**Labrador**	**4.5**	**5**	**3–5**
**B**	**Iceland**	**4.0**	**4**	**3–5**
	**Labrador**	**3.1**	**3**	**2–5**
**C**	**Iceland**	**2.7**	**3**	**2–3**
	**Labrador**	**3.5**	**3**	**2–5**

## Discussion

We conclude that *H*. *ampullatus* have a prolonged nursing period, based on a slow decrease in δ^15^N over GLGs 1–5. This decline was generally consistent across regions (N = 50 individuals) and between sexes (N = 48 individuals) and based on a chronological analysis of 39 individuals we found that weaning ends on average between ages three and four. Extended maternal care has not previously been documented in a beaked whale species and is in contrast to the only other estimate for *H*. *ampullatus* completing nursing in their first year, which was based on the stomach contents for a single calf [22]. This new evidence of extended care in *H*. *ampullatus* has implications for the life history and energetics of other species of beaked whales, as well as their ability to recover from the effects of whaling or other population level impacts such as disease or mass stranding events due to mid-frequency active sonar (MFAS) [37].

While the nursing duration varies widely across mammal taxa, it is known to be generally related to maternal body size, as prolonged nursing helps fulfill the caloric requirements for growth of larger independent animals [3,38]. Weaning typically occurs when offspring reach a certain size, and while beaked whales have proportionally larger calves compared to other cetaceans [21], between birth and age five juvenile *H*. *ampullatus* almost double their length from three to six meters, with adult whales reaching 7–9 meters [20]. Although the calves of the largest odontocete, *P*. *macrocephalus*, are relatively smaller at birth, (~ 33% of maternal size), they have prolonged lactation and nursing (mean 36 months, range 2–13 years [25,39]), presumably to support their growth and development. Due to the large calf size of beaked whales and prior assumptions of their short nursing duration and inter-calf intervals, it has been suggested that their reproduction somewhat resembles the capital breeding energetics of baleen whales (e.g. [5,21]). Unlike beaked whales, however, baleen whales are bulk feeders able to ingest large amounts of food over short time periods [40], limited by life history attributes tied to the seasonal constraints of migration and ocean productivity, and have significantly higher average milk fat percent to support the rapid growth, development and weaning of their calves [3,38].

Although the composition of whale milk is poorly documented across species, odontocetes are generally known to have energetically less rich milk (mean fat = 24%) than baleen whales (mean fat = 33%) [3,38]. The only two records available for beaked whales suggest their milk fat % is even lower than average for odontocetes, based on single records of specimens of *H*. *ampullatus* (20%) and *Mesoplodon stejnegeri* (17%) [38]. However, milk energy output is not strictly based on fat composition, as solids (protein, sugars and ash or minerals) also contribute to total calories available for consumption. For the odontocetes where total milk energy output has been calculated (*P*. *macrocephalus*, *Kogia breviceps*, *Delphinus delphis*, [38]), it is notably low, comparable only to values found in primates, which are also known to have long lactations and extended periods of dependency. While data are not available to calculate the energetic output of *H*. *ampullatus* milk, similar to other medium to large odontocetes, we suggest that prolonged nursing contributes to the caloric demands of rapid juvenile growth in the first 3 to 5 years.

Beyond providing necessary nutrition, nursing in mammals serves multiple functions; cetacean calves depend on nursing for their thermoregulation in the conversion of high fat milk into blubber, and maternal proximity offers protection from predators, ongoing socialization, and other important learning opportunities such as foraging and migration routes [3]. Prolonged nursing and gradual weaning, as part of the transition to nutritional independence, could be a life history adaptation for odontocetes with complex foraging strategies, such as deep diving. Both the biological demands and technical skills of foraging at depth may require time for physiological development and social learning. Although Newsome *et al*.’s study [18] of *P*. *macrocephalus* GLGs, found a gradual decrease in δ^15^N over the first 5 years, indicative of prolonged nursing, depth-recording tags indicated 1-year old calves had the capacity to dive to depths and durations of adult whales [41]. Whether *H*. *ampullatus* calves are also capable of diving to depths recorded for adult whales (e.g. 800–1400 m, [42]) is currently unknown. However, as juvenile beaked whales are overrepresented in mass stranding events linked to naval sonar, Hooker *et al*. [43] suggested that other aspects of dive capacity such as body mass, lung volume, or endurance for repeated dives, may be developmentally limited. We do know that for many species with a single precocial offspring, their young are introduced to solid food early despite prolonged nursing [3]. Thus, the need for prolonged maternal care in deep divers may also relate to the technical, socially learned aspects of foraging at depth, such as prey identification, capture and coordination with conspecifics.

While most isotopic studies of ontogeny have focussed on differences in ^15^N, here we also observed a regular pattern of increasing δ^13^C values from GLG 1 to older GLGs, which we suggest is consistent with weaning physiology. Milk is rich in ^13^C-depleted lipids, which if they are being incorporated into proteins, would lead to nursing animals having lower δ^13^C values than adults [12,44,45]. Although the trend for carbon is consistent with our inferences of prolonged nursing and a gradual transition from milk to solid food, gradual enrichment in ^13^C has not always been observed in other studies of odontocetes (e.g. *D*. *leucas*, [16]; *Grampus griseus*, [17]). As juvenile *H*. *ampullatus* whales learn to forage deeper, the increase in δ^13^C may reflect increasing consumption of bentho-pelagic species, which would be expected to have higher δ^13^C values [46]. Baseline δ^13^C can also vary spatially with latitude [47], and if all individuals demonstrated an ontogenetic shift in distribution it could potentially cause an increase or decrease in δ^13^C observed in tissues (e.g. [48]). However, based on global ^13^C isoscapes models [49], the lower latitudes (< 40°) where substantial foraging would have to occur to influence their δ^13^C profile, are at least 20°south of northern Labrador and Iceland, and outside of the known southern limit for the range of this species.

The patterns we observed appear largely consistent across a large number of specimens, however as a result of only including teeth with clearly defined GLG structure, we accept that our estimate of nursing duration may be biased towards healthy individuals. It is possible that age at weaning completion could be underestimated if the individuals in the study were weaned earlier due to available resources, or overestimated if maternal investment was longer than average. As our primary dataset included animals of different ages with a range of birth years spanning 1944–1967 (i.e. over four decades) it is unlikely either of these factors biased our results. The distinct GLG δ^15^N and δ^13^C patterns in the two whales which stranded in Newfoundland suggest that both individuals weaned earlier than the other specimens (at age 1–2, Fig 3a). Although there is no clear understanding of the relationship between δ^13^C and poor health conditions such as disease in whales, blubber stores may be mobilized during starvation or fasting (e.g *Ursus americanus*, [50]), and ^13^C depleted lipids would be incorporated in incremental tissues such as dentine. A notably decreasing rather than increasing δ^13^C trend (Fig 3b) in the stranded male whale could reflect a longer period of physiological decline. Absolute isotopic values of carbon and nitrogen also suggest that the diet of Newfoundland whales was distinct from the specimens killed in Labrador and Iceland 30 years prior (Fig 5a and 5b). While we attempted to account for known climatic trends in δ^13^C (i.e. Suess effect) by adjusting our δ^13^C values, other temporal influences we cannot account for, such as other baseline isotope or other ecosystem shifts, may have occurred across the North Atlantic during the ~ 30-year period separating specimens. This highlights some of the challenges in using stranded animals of unknown health status and specimens from disparate time periods to make broad inferences on poorly understood species biology. Further investigation on the relationship between health status and the appearance of GLG structure in marine mammals would help clarify the influence of these factors for future studies.

Interestingly, our finding that δ^15^N in fetal dentine was almost 1 ‰ lower than GLG 1 differs from the pattern of steady decline in δ^15^N from a peak in fetal dentine observed in other species of cetaceans (*Grampus griseus*, [17]; *Monodon Monoceros*, Zhao *et al*. unpublished data; *Delphinapterus leucas*, Matthews & Zhao, unpublished data). Our explanations for the inconsistencies between enrichment patterns in fetal dentine across cetacean species consider two possibilities: (1) if tissues measured in other studies are actually neonatal rather than fetal dentine, δ^15^N for other species would reflect an ongoing decline in post-partum nursing [51]; or (2) differences are due to species-specific reproductive biology, such as physiological differences between capital and income breeders or growth dependent trophic enrichment factors. While occasional errors in identification of fetal dentine may occur, as Stewart & Stewart [52] describe there are multiple established landmarks for distinguishing pre and post-natal dentine deposition, making it unlikely that this is the source of consistent error across studies. Borrell *et al*. [12] found fetal tissues of capital breeders, which sustain reproduction with stored fat reserves, were higher in δ^15^N than their mothers, whereas for income breeders, mother-fetus δ^15^N discrimination was not observed. While odontocetes are generally recognized as income breeders, as per Huang *et al*. [21], aspects of *H*. *ampullatus* prenatal reproductive energetics, such as large relative calf size, do not align with the other odontocete species. Alternatively, if growth dependent ^15^N enrichment occurs due to rapid development in utero, it could explain fetal δ^15^N patterns, which may be different in smaller cetacean species than for larger species such as *H*. *ampullatus*. The inconsistencies in fetal development between species highlight the need to better understand the influence of maternal physiology on fetal development and stable isotope discrimination so that future studies can accurately interpret stable isotope profiles [12].

Theory predicts that parents in polygynous species may adopt a sex-bias in infant investment towards males [53]. While we do not have a good understanding of the mating systems across any of the species of beaked whales [54], most are sexually dimorphic, and in *H*. *ampullatus*, males are significantly larger in size, suggesting they need additional energetic resources for growth [55]. Although Hooker *et al*. [56] found adult males were marginally enriched in ^15^N relative to females, we did not find significant evidence that this occurs as part of maternal investment. While there may be some influence of sex on trophic position in mature animals, there was no difference between males and females in terms of nursing duration, or relative values of δ^15^N or δ^13^C across GLGs. However individual variation and annual averaging within GLGs may mask the presence of finer scale sex-based patterns or trends in isotopic enrichment (Figs 3a and 4) [57].

The weaning period, which includes the introduction to solid food accompanied by nursing, can vary in length depending on whether maternal weaning strategies are abrupt or gradual. Using the timing associated with the cessation of a general declining trend in δ^15^N, changepoint analysis or model fit against a number of theoretical curves, a number of authors [14,16,17] have proposed that unlike baleen whales, weaning in odontocetes is a relatively gradual process. While the introduction of solid food may occur within the first year, this is often accompanied by prolonged nursing across a number of odontocete species [4], suggesting stomach contents are unlikely to provide good evidence of the age when weaning is complete. The point when δ^15^N values become relatively stable and more consistent with subsequent GLGs or are approximately equivalent to mature baseline values has been used to estimate weaning completion [14,16,51]. For *H*. *ampullatus*, we found generally similar results across methods, suggesting weaning was complete when whales were between 3 to 4 years old. The point when δ^15^N values stopped decreasing (Method A), suggested δ^15^N declined into year five for some individuals, which could reflect individual variation in prolonged nursing, or differences in ability to forage on higher trophic level prey. Defining weaning completion as the point when δ^15^N was equal to mature values (Method B), or when δ^15^N was 1.2‰ lower than GLG1 (Method C), suggests that in *H*. *ampullatus*, similar to other odontocetes, nursing is prolonged with weaning taking over 3 years to complete.

If Benjaminsen [22] was correct in their calculation of a 12 month gestation period for *H*. *ampullatus*, nursing a calf for at least 3 years would double previous estimates of their reproductive cycle to at least 4 years [20]. New *et al*.’s [5] bioenergetic models of beaked whales found that low survival and reproduction was tied to the relatively short estimates for duration of lactation, and the assumption of a 2-year calving interval. Energetically, a large percentage of beaked whales in New *et al*.’s [5] models had difficulty meeting their metabolic requirements under standard assumptions and inferred reproductive parameters derived from historic whaling data. Prolonged nursing was identified by New *et al*. [5] as an alternate strategy that would give females a recovery period between mating, allowing them to rebuild energetic stores and increase the probability of their next calf’s survival. Prolonged maternal investment and a longer inter-calving interval also has consequences for the rate of effective population growth. Given the assumption that for most odontocetes, pregnancy and lactation rarely overlap, extended nursing decreases the lifetime reproductive potential of the species by half. For *H*. *ampullatus*, extended maternal care would prolong their recovery from commercial whaling and increase the impact of contemporary risks to their populations such as disease outbreaks, MFAS induced strandings or other unusual mortality events [3–5,37,58,59].

A longer nursing period also implies that *H*. *ampullatus* have extended maternal associations, and suggests that social structure of beaked whales may be more complex than previous observational studies have been able to detect [54,60]. Generally beaked whales are found in very small groups and are not considered particularly social, however in well studied beaked whales (e.g. *Ziphius cavirostris*, *Berardius sp*., *Mesoplodon densirostris)*, there is some evidence of long-term bonds (over months to years) between individuals using photo-ID methods [19,54]. Although McSweeney [61] documented repeated associations over two years between a female *Ziphius cavirostris* and her calf, and Baird [54] suggests that *M*. *densirostris* calves disperse from their mothers between 2–3 years of age, long-term associations with relatively unmarked beaked whale calves are particularly hard to track using photo-identification. In the only study where putative mother-calf relationships were assessed in *H*. *ampullatus*, repeated associations over two subsequent years were only documented twice [60,62]. From our review, the range of estimates for the duration of lactation, weaning period, age of dispersal or inter-calf interval in beaked whales has either been inferred from the maximum length of maternal-calf associations using photo-identification analysis or applied across species using limited stomach content data (e.g. see [5]). Thus, our study provides the first significant dataset for interpreting the range of variation in individual maternal investment in a species of beaked whale and improves our understanding of the diversity in maternal strategies found across cetaceans and mammals.

## Supporting information

S1 Table Summary of cetacean studies reporting nursing duration or weaning age by species, method, average age at weaning, and sample type. (DOCX)

S2 Table Rationale for inclusion of variables and data sources. (DOCX)

## References

[1] Rendell L, Cantor M, Gero S, Whitehead H, Mann J. Causes and consequences of female centrality in cetacean societies. Phil Trans R Soc B. 2019;374: 20180066–13. 10.1098/rstb.2018.0066 31303160 PMC6664132

[2] Clutton-Brock T. Mammal Societies. Chichester: John Wiley & Sons; 2016.

[3] Hayssen V, Orr TJ. Reproduction in mammals: the female perspective. Baltimore: Johns Hopkins University Press; 2017.

[4] Whitehead H, Mann J. Female reproductive strategies of cetaceans, life history and calf care In: Mann J, Connor RC, Tyack PL, Whitehead H, editors. Cetacean societies: field studies of dolphins and whales. Chicago: University of Chicago Press; 2000.

[5] New LF, Moretti DJ, Hooker SK, Costa DP, Simmons SE. Using Energetic Models to Investigate the Survival and Reproduction of Beaked Whales (family Ziphiidae). PLoS ONE. 2013;8: e68725–14. 10.1371/journal.pone.0068725 23874737 PMC3714291

[6] Trivers R. Parental investment and sexual selection In: de Gruyter A, editor. Sexual Selection & the Descent of Man. New York: Aldine Publishing Company; 1972.

[7] Lee P.C., Majluf P, Gordon IJ. Growth, weaning and maternal investment from a comparative perspective. J Zool. 1991;225: 99–114.

[8] Clutton-Brock TH, Albon SD, Guinness FE. Parental investment in male and female offspring in polygynous mammals. Nature. 1981;289: 487–489. 10.1038/289487a0

[9] Clutton-Brock TH. Mammalian mating systems. Proc R Soc B. 1989;236: 339–372. 10.1098/rspb.1989.0027 2567517

[10] Trivers R. Parent-offspring conflict. Am Zool. 1974;14: 249–264.

[11] Perrin WF, Reilly S. Reproductive parameters of dolphins and small whales of the family Delphinidae. Report of the International Whaling Commission. 1984;6: 97–133.

[12] Borrell A, Gómez-Campos E, Aguilar A. Influence of reproduction on stable-isotope ratios: nitrogen and carbon isotope discrimination between mothers, fetuses, and milk in the fin whale, a capital breeder. Physiol Biochem Zool. University of Chicago PressChicago, IL; 2015;89: 41–50. 10.1086/684632 27082523

[13] Mendes S, Newton J, Reid RJ, Zuur AF, Pierce GJ. Stable carbon and nitrogen isotope ratio profiling of sperm whale teeth reveals ontogenetic movements and trophic ecology. Oecologia. 2007;151: 605–615. 10.1007/s00442-006-0612-z 17124567

[14] Newsome SD, Etnier MA, Monson DH, Fogel ML. Retrospective characterization of ontogenetic shifts in killer whale diets via δ^13^C and δ^15^N analysis of teeth. Mar Ecol Prog Ser. 2009;374: 229–242. 10.3354/meps07747

[15] Rossman S, Ostrom PH, Stolen M, Barros NB, Gandhi H, Stricker CA, et al Individual specialization in the foraging habits of female bottlenose dolphins living in a trophically diverse and habitat rich estuary. Oecologia. 2015;178: 415–425. 10.1007/s00442-015-3241-6 25656585

[16] Matthews CJD, Ferguson SH. Weaning age variation in beluga whales (*Delphinapterus leucas*). J Mammal. 2015;96: 425–437. 10.1093/jmammal/gyv046

[17] Evacitas FC, Kao WY, Worthy GAJ, Chou LS. Annual variability in dentin δ^15^N and δ^13^C reveal sex differences in weaning age and feeding habits in Risso’s dolphins (*Grampus griseus*). Marine Mammal Sci. 2017;33: 748–770. 10.1111/mms.12396

[18] Newsome SD, Clementz MT, Koch PL. Using stable isotope biogeochemistry to study marine mammal ecology. Marine Mammal Sci. 2010;52: 837–64. 10.1111/j.1748-7692.2009.00354.x

[19] Hooker SK, de Soto NA, Baird RW, Carroll EL, Claridge D, Feyrer L, et al Future directions in research on beaked whales. Front Mar Sci. 2019;6: 217 10.3389/fmars.2018.00514

[20] Benjaminsen T, Christensen I. The natural history of the bottlenose whale, *Hyperoodon ampullatus* In: Winn HE, editor. Behavior of Marine Animals. New York: Plenum Press; 1979 pp. 143–164.

[21] Huang S-L, Chou LS, Shih N-T, Ni I-H. Implication of life history strategies for prenatal investment in cetaceans. Marine Mammal Sci. 2010;27: 182–194. 10.1111/j.1748-7692.2010.00392.x

[22] Benjaminsen T. On the Biology of the Bottlenose Whale, *Hyperoodon ampullatus* (Forster). Norweg J Zool. 1972;20: 233–241.

[23] Scheffer VB, Myrick A. A review of studies to 1970 of growth layers in the teeth of marine mammals. Report of the International Whaling Commission. 1980;3: 51–63.

[24] Ellis R, Mead JG. Beaked Whales: A Complete Guide to their Biology and Conservation. Baltimore: John Hopkins University Press; 2017.

[25] Gero S, Gordon J, Whitehead H. Calves as social hubs: dynamics of the social network within sperm whale units. Proc R Soc B. 2013;280: 20131113–9. 10.1098/rspb.2013.1113 23740785 PMC3774244

[26] Christensen I. Age determination, age distribution and growth of bottlenose whales, *Hyperoodon ampullatus* (Forster), in the Labrador Sea. Norweg J Zool. 1973;21: 331–340.

[27] Einfeldt AL, Orbach DN, Feyrer LJ. A method for determining sex and chromosome copy number: sex-by-sequencing reveals the first two species of marine mammals with XXY chromosome condition. J Mammal. 2019;35: 1228–7. 10.1093/jmammal/gyz131

[28] Feyrer LJ, Bentzen P, Whitehead H, Paterson IG, Einfeldt A. Evolutionary impacts differ between two exploited populations of northern bottlenose whale (*Hyperoodon ampullatus*). Ecol Evol. 2019;1: 101–18. 10.1002/ece3.5813 31871667 PMC6912904

[29] Klevezal GA. Recording structures of mammals: Determination of age and reconstruction of life history Recording Structures of Mammals: Determination of Age and Reconstruction of Life History. Rotterdam; 1996 pp. 1–274.

[30] Luque SP, Higdon JW, Ferguson SH. Dentine deposition rates in belugas (*Delphinapterus leucas*): An analysis of the evidence. Aquat Mamm. 2007;33: 241–245. 10.1578/AM.33.2.2007.241

[31] Read FL, Hohn AA, Lockyer CH. A review of age estimation methods in marine mammals with special reference to monodontids. NAMMCOSP. 2018;10: 1–67. 10.7557/3.4474

[32] Pierce KV, Kajimura H. Acid etching and highlighting for defining growth layers in cetacean teeth. Report of the International Whaling Commission. 1980;3: 99–103.

[33] Prolonged maternal investment in northern bottlenose whales alters our understanding of beaked whale reproductive life history. Dryad; 2020. 10.5061/dryad.k98sf7m3j PMC7310684 32574188

[34] Quay P, Sonnerup R, Westby T, Stutsman J, McNichol A. Changes in the ^13^C/^12^C of dissolved inorganic carbon in the ocean as a tracer of anthropogenic CO^2^ uptake. Global Biogeochemical Cycles. John Wiley & Sons, Ltd; 2003;17: 1–20. 10.1029/2001GB001817

[35] English PA, Green DJ, Nocera JJ. Stable Isotopes from Museum Specimens May Provide Evidence of Long-Term Change in the Trophic Ecology of a Migratory Aerial Insectivore. Front Ecol Evol. 2018;6: 262 10.3389/fevo.2018.00014

[36] Bates D, Maechler M, Bolker B, Walker S. lme4: Linear mixed-effects models using Eigen and S4: R package version 1.1–7.

[37] Simonis AE, Brownell RL Jr, Thayre BJ, Trickey JS, Oleson EM, Huntington R, et al Co-occurrence of beaked whale strandings and naval sonar in the Mariana Islands, Western Pacific. Proc R Soc B. 2020;287: 20200070–10. 10.1098/rspb.2020.0070 32070257 PMC7062028

[38] Oftedal OT. Lactation in whales and dolphins: evidence of divergence between baleen- and toothed-species. J Mammary Gland Biol Neoplasia. 1997;2: 205–230. 10.1023/a:1026328203526 10882306

[39] Best PB, Canham PA, Macleod N. Patterns of reproduction in sperm whales, *Physeter macrocephalus*. Report of the International Whaling Commission. 1984;6: 51–79.

[40] Goldbogen JA, Cade DE, Calambokidis J, Friedlaender AS, Potvin J, Segre PS, et al How Baleen Whales Feed: The Biomechanics of Engulfment and Filtration. Annu Rev Mar Sci. 2017;9: 367–386. 10.1146/annurev-marine-122414-033905 27620830

[41] Tønnesen P, Gero S, Ladegaard M, Johnson M, Madsen PT. First-year sperm whale calves echolocate and perform long, deep dives. Behav Ecol Sociobiol. Springer Berlin Heidelberg; 2018;72: 201–15. 10.1007/s00265-018-2570-y

[42] Hooker SK, Baird RW. Deep–diving behaviour of the northern bottlenose whale, *Hyperoodon ampullatus* (Cetacea: Ziphiidae). Proc R Soc B. The Royal Society; 1999;266: 671–676. 10.1098/rspb.1999.0688

[43] Hooker SK, Baird RW, Fahlman A. Could beaked whales get the bends? Effect of diving behaviour and physiology on modelled gas exchange for three species: *Ziphius cavirostris*, *Mesoplodon densirostris* and *Hyperoodon ampullatus*. Respir Physiol Neurobiol. 2009;167: 235–246. 10.1016/j.resp.2009.04.023 19427415

[44] DeNiro MJ, Epstein S. Mechanism of carbon isotope fractionation associated with lipid synthesis. Science. 1977;197: 261–263. 10.1126/science.327543 327543

[45] Newsome SD, Koch PL, Etnier MA, Aurioles-Gamboa D. Using carbon and nitrogen isotope values to investigate maternal strategies in northeast pacific otariids. Marine Mammal Sci. 2006;22: 556–572. 10.1111/j.1748-7692.2006.00043.x

[46] Trueman CN, Johnston G, O'Hea B, MacKenzie KM. Trophic interactions of fish communities at midwater depths enhance long-term carbon storage and benthic production on continental slopes. Proc R Soc B. 2014;281: 20140669–20140669. 10.1098/rspb.2014.0669 24898373 PMC4071550

[47] Trueman CN, St John Glew K. Isotopic tracking of marine animal movement In: Hobson KA, Wassenaar LI, editors. Tracking Animal Migration with Stable Isotopes. Second Edition Elsevier Inc; 2018 pp. 137–172.

[48] Trueman CN, Jackson AL, Chadwick KS, Coombs EJ, Feyrer LJ, Magozzi S, et al Combining simulation modeling and stable isotope analyses to reconstruct the last known movements of one of Nature’s giants. PeerJ. 2019;7: e7912–20. 10.7717/peerj.7912 31637141 PMC6802580

[49] Magozzi S, Yool A, Vander Zanden HB, Wunder MB, Trueman CN. Using ocean models to predict spatial and temporal variation in marine carbon isotopes. Ecosphere. 2nd ed 2017;8: e01763–20. 10.1002/ecs2.1763

[50] Ahlquist DA, Nelson RA, Steiger DL, Jones JD, Ellefson RD. Glycerol metabolism in the hibernating black bear. J Comp Physiol B. 1984;155: 75–79. 10.1007/BF00688794

[51] Riofrío-Lazo M, Aurioles-Gamboa D, Le Boeuf BJ. Ontogenetic changes in feeding habits of northern elephant seals revealed by δ^15^N and δ^13^C analysis of growth layers in teeth. Mar Ecol Prog Ser. 2012;450: 229–241. 10.3354/meps09562

[52] Stewart BE, Stewart RE. The biology behind the counts: tooth development related to age estimation in beluga (*Delphinapterus leucas*). NAMMCOSP. 2010;8: 1–20. 10.7557/3.3195

[53] Maynard Smith J. A new theory of sexual investment. Behav Ecol Sociobiol. Springer-Verlag; 1980;7: 247–251. 10.1007/BF00299371

[54] Baird RW. Behavior and Ecology of Not-So-Social Odontocetes: Cuvier’s and Blainville’s Beaked Whales In: Wursig B, editor. Ethology and Behavioral Ecology of Odontocetes Ethology and Behavioral Ecology of Marine Mammals. Cham: Springer International Publishing; 2019 pp. 305–329.

[55] Clutton-Brock TH, Albon SD, Guinness FE. Parental investment and sex differences in juvenile mortality in birds and mammals. Nature. 1985;313: 131–133.

[56] Hooker SK, Iverson SJ, Ostrom P, Smith SC. Diet of northern bottlenose whales inferred from fatty-acid and stable-isotope analyses of biopsy samples. Can J Zool. 2001;79: 1442–1454. 10.1139/z01-096

[57] Smith K, Sparks J, Timmons ZL, Peterson MJ. Cetacean skeletons demonstrate ecologically relevant variation in intraskeletal stable isotopic values. Front Mar Sci. 2020;7: 388.

[58] Grove T, Senglat C, Petitguyot M, Kosiba D, Rasmussen MH. Mass stranding and unusual sightings of northern bottlenose whales (*Hyperoodon ampullatus*) in Skjálfandi Bay, Iceland. Marine Mammal Sci. 2020;2018: 227–9. 10.1111/mms.12689

[59] Barley J, Foster G, Reid B, Dagleish M, Howie F. Encephalitis in a northern bottlenose whale. Vet Rec. 2007;160: 452–452. 10.1136/vr.160.13.452-a 17400910

[60] Gowans S, Whitehead H, Hooker SK. Social organization in northern bottlenose whales, *Hyperoodon ampullatus*: not driven by deep-water foraging? Anim Behav. 2001;62: 369–377. 10.1006/anbe.2001.1756

[61] McSweeney DJ, Baird RW, Mahaffy SD. Site fidelity, associations, and movements of Cuvier’s (*Ziphius cavirostris*) and Blainville’s (*Mesoplodon densirostris*) beaked whales off the island of Hawai’i. Marine Mammal Sci. 2007;23: 666–687.

[62] Gowans SE. Social organization and population structure of northern bottlenose whales in the Gully. Dalhousie University 1999 pp. 1–245. https://dalspace.library.dal.ca/.

