# Supplementary material for: Prolonged maternal investment in northern bottlenose whales alters our understanding of beaked whale reproductive life history
Source: PLoS One. 2020 Jun 23;15(6):e0235114. doi: 10.1371/journal.pone.0235114 (PMC7310684; doi:10.1371/journal.pone.0235114)
Supplement: S1 Table — (DOCX) [file pone.0235114.s001.docx]

**S1 Table. Summary of cetacean studies reporting nursing duration or weaning age by species, method, average age at weaning, and sample type.**

| Common name | Genus species | Method | N | Weaning age  mean months | Range  months | Oldest weaned  age | Sample Type | Year | Reference |
| --- | --- | --- | --- | --- | --- | --- | --- | --- | --- |
| 10 Mysticete species: Blue, Fin, Sei, Minke, Humpback, Bowhead, Pygmy Right, Gray, Bryde | B. musculus, B. physalus, B. borealis, B. brydei, B. acutorostrata, B.novaeangliae, B. mysticetus, C. marginata, E. robustus | Cow-calf/ Stomach contents | n.r. | 8 | 4-12 |  | Hunted animals | 1984 | Lockyer 1984 |
| Commerson's dolphin | Cephalorhynchus c. commersonii | δ13C and δ15N -bone collagen | 220 | 6 | 6-12 | n.r. | Stranded/ bycatch | 2013 | Riccialdelli et al. 2013 |
| Beluga | Delphinapterus leucas | δ15N of tooth dentin GLGs | 25 | 30 | 2-3 years | 4 yrs | Hunted animals | 2015 | Matthews & Ferguson 2015 |
| Short-beaked common dolphins | Delphinus delphis | Teeth structure - accessory lines | 117 | 18 | 1-2 years | n.r. | Wild population | 2013 | Luque et al. 2013 |
| Common dolphins | Delphinus delphis | n.r. | n.r. | 5.5 | 5-6 | n.r. | Hunted animals | 1984 | Reported in Perrin and Reilly 1984 |
| South Atlantic Right whales | Eubalaena australis | Behaviour | 16 | 13 | 12-14 | 14 months | Wild population | 1982 | Taber & Thomas 1982 |
| North Atlantic Right whales | Eubalaena glacialis | Behaviour | 22 | 12 |  | 14 months | Wild population | 2010 | Hamilton & Cooper 2010 |
| Short-finned pilot whale | Globicephala macrorhynchus | Stomach contents/ Cow-calf method | n.r. | 36 | 3.5-5 years | 13 years | Hunted animals | 1984 | Kasuya & Marsh 1984 |
| Short-finned pilot whale | Globicephala macrorhynchus | Stomach contents/ Cow-calf method | 31 | 9 |  | 22 months | Drive fishery | 1962 | Sergeant 1962 |
| Long-finned Pilot whales | Globicephala melas | Stomach contents | n.r. | 22 |  |  | Hunted animals | 1962 | Sergeant 1962 |
| Rissos Dolphin | Grampus griseus | δ15N of tooth dentin GLGs | 22 | 17.4 |  | 2.13 years | Stranded/ bycatch | 2017 | Evacitas et al. 2017 |
| Rissos Dolphin | Grampus griseus | Cow-calf method | 16 | 24 |  | 3 years | Drive hunted | 2012 | Bloch et al. 2012 |
| Rissos Dolphin | Grampus griseus | Cow-calf method | 21 | 18 | 1-1.6 years | 3 years | Drive hunted | 2004 | Amano & Miyazaki 2004 |
| Northern bottlenose whale | Hyperoodon ampullatus | Stomach contents | n.r. | 12 |  | n.r. | Hunted animals | 1979 | Benjaminsen & Christensen 1979 |
| Killer whale | Orcinus orca | δ15N of tooth dentin GLGs | 11 | 42 | 3- 4 years |  | Stranded animals | 2009 | Newsome et al. 2009 |
| Killer whale | Orcinus orca | Behaviour | n.r. | 18 |  | n.r. | Captive born | 1999 | Dahlheim & Heyning 1999 |
| Killer whale | Orcinus orca | Behaviour |  | >12 |  |  | Wild | 1982 | International Whaling Commission 1982 |
| Killer whale | Orcinus orca | Behaviour | n.r. | 24 |  | n.r. | Wild population | 1979 | Dahlheim & Heyning 1979 |
| Dall's porpoise | Phocoena dalli | Stomach contents | n.r. | 24 | 6-36 | 3 years | Hunted animals | 1999 | Ferrero & Walker 1999 |
| Harbour porpoise | Phocoena phocoena | Teeth structure - accessory lines | 167 | 12 |  | 3 years | Stranded animals | 2009 | Luque et al. 2009 |
| Harbour porpoise | Phocoena phocoena | Behaviour | 1 | 9 | 8-10 |  | Live capture | 2003 | Lockyer & Kinze 2003 |
| Sperm whale | Physeter macrocephalus | Stomach contents | 27 | 7.5 years | 2-13 years | 13 yrs | Catch data | 1984 | Best et al. 1984 |
| Franciscana | Pontoporia blainvillei | δ15N of dorsal muscle tissue/ stomach contents | 54 | 12 |  | 2 years | Bycatch | 2017 | Viola et al. 2017. |
| Franciscana | Pontoporia blainvillei | δ15N of whole tooth | 125 | 48 |  |  | Bycatch | 2016 | Troina et al. 2016 |
| Franciscana | Pontoporia blainvillei | Stomach contents | 26 | 7 |  |  | Bycatch, calves | 2013 | Denuncio et al. 2013 |
| Franciscana | Pontoporia blainvillei | Stomach contents | 110 | 7 |  |  | Bycatch | 2002 | Rodríguez et al. 2002 |
| False killer whales | Pseudorca crassidens | δ13C and δ15N bone collagen |  | 23 | 19-24 |  | Stranded animals | 2015 | Riccialdelli & Goodall 2015 |
| False killer whales | Pseudorca crassidens | Behaviour | 2 | 24 |  | 2 years | Captive born | 1999 | Clark & Odell 1999 |
| Guianna dolphin | Sotalia guianensis | Teeth structure - accessory lines | 71 | 8 | 6.7 - 10.3 |  | Bycatch | 2003 | Rosas et al. 2003 |
| Pantropical spotted dolphin | Stenella attenuata | Stomach contents | 203 | 9 |  | 2 years | Bycatch | 2004 | Archer & Robertson 2004 |
| Pantropical spotted dolphin | Stenella attenuata | Cow-calf method | n.r. | 20 |  | 60 months | Hunted animals | 1984 | Reported in Perrin and Reilly 1984 |
| Striped dolphin | Stenella coeruleoalba | Cow-calf method | n.r. | 16 |  | 36 months | Hunted animals | 1984 | Reported in Perrin and Reilly 1984 |
| Striped dolphin | Stenella coeruleoalba | Cow-calf method/ Stomach contents | 45 | 18 | 3 yrs | n.r. | Drive fishery | 1977 | Miyazaki 1977 |
| Spinner dolphin | Stenella longirostris | Cow-calf method | n.r. | 21 |  | 34 months | Hunted animals | 1984 | Reported in Perrin and Reilly 1984 |
| Indo-pacific bottlenose dolphins | Tursiops aduncus | Behaviour | 22 | 42 |  | 6 yrs | Wild population | 2004 | Kogi et al. 2004. |
| Bottlenose dolphins | Tursiops sp. | Behaviour | 74 | 48 |  | 8 years | Wild population | 2000 | Mann et al. 2000 |
| Bottlenose dolphins | Tursiops sp. | Behaviour | summary | 19 | 18-20 | 38 months | Wild and captive | 1984 | Reported in Perrin & Reilly 1984 |
| Bottlenose dolphins | Tursiops truncatus | Behaviour | 11 | 36 |  | 4 yrs | Wild population | 2018 | Baker et al. 2018 |
| Bottlenose dolphins | Tursiops truncatus | Behaviour | 136 | 47 |  | 8.59 yrs | Wild population | 2018 | Karniski et al. 2018 |
| Bottlenose dolphin | Tursiops truncatus | δ15N - tooth dentin age classes | 15 | 36 | 2-4 years |  | Stranded animals (female) | 2015 | Rossman et al. 2015 |
| Bottlenose dolphins | Tursiops truncatus | δ13C and δ15N - whole teeth | 49 | 24 |  | n.r. | Stranded animals (age 1-5) | 2015 | Fruet et al. 2015 |
| Bottlenose dolphins | Tursiops truncatus | δ 13C and δ 15N - teeth and skin samples | 60/32 | 24 |  | 3 years | Stranded animals | 2008 | Knoff et al. 2008 |
| Bottlenose dolphins | Tursiops truncatus | n.r. | n.r. | 12 |  | 7 years | Wild population | 1999 | Wells et al. 1999 |
| Bottlenose dolphins | Tursiops truncatus | Stomach contents | n.r. | 12 |  | 3 years | Stranded/ bycatch | 1990 | Cockcroft & Ross 1990 |
