# Supplementary material for: Prolonged maternal investment in northern bottlenose whales alters our understanding of beaked whale reproductive life history
Source: PLoS One. 2020 Jun 23;15(6):e0235114. doi: 10.1371/journal.pone.0235114 (PMC7310684; doi:10.1371/journal.pone.0235114)
Supplement: S2 Table — (DOCX) [file pone.0235114.s002.docx]

S2 Table. Variable inclusion rationale and data sources

| **Variable** | **Study objective** | **Study Question** | **Data Source** |
| --- | --- | --- | --- |
| δ^15^N | Weaning Age | Estimate trophic level of foraging, variation over time, end of weaning period | GG Hatch Lab |
| δ^13^C | Foraging Behaviour | Estimate baseline primary productivity between regional ecosystems and weaning related influences | GG Hatch Lab |
| Location | Weaning Age  Foraging Behaviour | Identify population level or ecosystem differences in weaning or foraging behaviour | Lat/Long from whaling records |
| GLG - Year | Weaning Age | Identify fine scale differences over the first five years | Counts from tooth specimens |
| Sex | Weaning Age  Foraging Behaviour | Identify sex bias in maternal provisioning and foraging behaviour | Genetic analysis |
| Individual | Weaning Age | Identify between and within individual variation | Whaling records |
