# Supplementary material for: Prolonged maternal investment in northern bottlenose whales alters our understanding of beaked whale reproductive life history
Source: PLoS One. 2020 Jun 23;15(6):e0235114. doi: 10.1371/journal.pone.0235114 (PMC7310684; doi:10.1371/journal.pone.0235114)
Supplement: Attachment — Submitted filename: Response to reviewers.docx [file pone.0235114.s003.docx]

| Editor | the PLOS ONE style templates can be found at  https://journals.plos.org/plosone/s/file?id=wjVg/PLOSOne_formatting_sample_main_body.pdf and  https://journals.plos.org/plosone/s/file?id=ba62/PLOSOne_formatting_sample_title_authors_affiliations.pdf | Yes | Corrected as per the style guide provided |
| --- | --- | --- | --- |
| Editor | the DOI address provided does not show data. include the relevant URLs, DOIs, or accession numbers within your revised cover letter. | Yes | The temporary link to the data on dryad is: <https://datadryad.org/stash/share/lUjjQbtb6qJD2Dmg7YC8_0kHghanCrufbdiPwusgnWw>.  The public DOI for the data on dryad is doi:10.5061/dryad.k98sf7m3j  but it will not work until the paper is accepted and has been approved by dryad currators |
| Editor | We note that Figure 1 in your submission contains map/satellite images which may be copyrighted. | NA | L. Feyrer created this map in R using publicly accessible bathymetric data, holds the copyright, and gives permission for use of this image. |
| Editor | Please include captions for your Supporting Information files at the end of your manuscript, and update any in-text citations to match accordingly. | Yes | Corrected as per the style guide provided |
| Editor | Clarify the sample sizes: In the introduction (line 105), 151 teeth are noted (from 151 individuals?), while in the methods (line 126 and 130), 50 is noted (from 50 individuals?), as on line 144 ('only 50'). Later (line 184) this 50 is reduced to a sub-set of 39, although it is unclear why the 50 with GLGs1-5 were reduced to those with GLGs1-3? Please examine the original sample (n = 151 individuals) and walk the readers through the steps taken to reach the final (n = 50 teeth) sample size. | Yes | Clarified where n = individuals and the rationale/ process for initial screening reducing sample size to 50 ID's for overall analysis (starting at Ln 160) Then due to the criteria for GLG weight (ln 200-210), some IDs did not have all GLGs from 1-3 available (N = 39). Revised Table 1 to further clarify. |
| Editor | Similarly, the discussion (line 302) notes "across 50 individuals", but based on Figure 3 it seems this should be 39? | Yes | This section of the methods and table 1 (lns 211) has been amended to clarify which analyses and how many IDs are being discussed as the trends by region / sex / ontogenetic analyses included data from different #'s of individuals. |
| Editor | In Figure 4 the sample size values for males and females should be explained in the caption, as these could easily be construed as the number of individuals (which presumably they are not). | Yes | Revised figure caption as suggested |
| Editor | - In Table 1, please clarify the following numbers: n=288/244, n=207, n=124, n=112, n=110, n=97 | Yes | Revised Table 1 to clarify sample size for individuals and GLG samples overall and by region. Omitted sample size breaks down by sex as it is described in the figures and slightly different for each analysis. |
| Editor | - In Figure 4 a-b and 5a-b. Report the sample size for each point, not the total. | Yes | Revised figures as suggested |
| Editor | In Table 2. Sort based on increasing AIC values. | Yes | reordered by decreasing AIC |
| Editor | NULL model - You should include it to your list: d15N~1+(1\|Tooth_N) | Yes | Included as suggested |
| Editor | - In Table 3. Modify italic font to bold on the first line of Table 3a. | Yes | Revised as suggested. |
| Editor | - In addition, modify 0.000 entries by >0.001 for very small p-values. | Yes | Revised to < 0.001 as this is assumed to be what the reviewer was suggesting. |
| Editor | - In Lines 287-288 and Table 4.  Be consistent with number of decimal places shown for these values. | Yes | Revised as suggested. |
| Editor | In Lines 113, 320, 333, 337, 367, 404. Make sure the use of the common and scientific names is consistent. Currently, sometimes they are both used, sometimes only one or the other are used, … | Yes | Revised throughout to use primarily scientific species names for simplicity |
| Editor | - Line 470. This discussion could be broadened to include population recovery in response to other impacts (e.g., MFAS, disease outbreaks). | Yes | Amended to include these population level impacts |
| Reviewer 1 | l.182: change the font of | Yes | corrected as per the style guide provided |
| Reviewer 1 | l.184: n=39 or n=37 as appears in Table 1? | Yes | N = 39 |
| Reviewer 1 | Table 1: n=288/244, n=207, n=124, n=112, n=110, n=97: what are those numbers? Could you please specify? | Yes | specified and amended Table 1 |
| Reviewer 1 | Fig 4a-b and 5a-b: You should give the sample size for each point, not the total. Otherwise it is confusing. | Yes | Revised figures as suggested |
| Reviewer 1 | Table 2: I do not see the results for the null model in your candidate models? You should include it to your list: d15N~1+(1\|Tooth_N). | Yes | Added null model to table 2 a, b. not significant |
| Reviewer 1 | Table 3: Modify italic font to bold on the first line of Table 3a. In addition, you should modify 0.000 entries by >0.001 for very small p-values. | Yes | Revised to < 0.001 as this is assumed to be what the reviewer was suggesting. |
| Reviewer 2 | I did not see a Data Availability Statement with the manuscript. | Yes | It was included in the original submission form, but it has now been added as a section in the manuscript although from the guidelines it is unclear whether this is where it is required. |
| Reviewer 2 | Beaked whales are a speciose group, and this study focuses on only one species. I'd suggest either discussing what is known of weaning age in other well-studied beaked whales (Berardius, Ziphius, M. densirostris) or narrowing the focus on the title to "... northern bottlenose whale reproductive life history". For species that do not have long-term bonds (e.g., Ziphius, M. densirostris) the age of dispersal of an offspring away from its mother would also be a good indicator of maximum age of weaning. It would be good to note whether any information is available on this from other species of beaked whales that have been the subject of photo-ID studies. Some reference (in lines 304-308) to what is known (or not known) about northern bottlenose whale disassociations between mothers and calves based on photo-ID would be warranted. | Yes | Added a new paragraph at the end of the discussion that references other records of age of dispersal and association length between mother-calf pairs, including NBW and limited data from photo ID studies. Added NBW to the title for clarity. |
| Reviewer 2 | Line 27, 136, 483. The term "whaled" is used instead of "hunted". Would be more explicit to use hunted. | Yes | Used hunted where it was a general reference and whaled in specific reference to our dataset which was collected by commercial whalers, not aboriginal or other hunters |
| Reviewer 2 | Line 43. Use "factors" instead of "constraints"? | Yes | revised as suggested. |
| Reviewer 2 | Lines 100-103. The sentence "However beaked whales are...." is not particularly relevant and not a good transition sentence for introducing the purpose of this study. That they only have two teeth or that the teeth only erupt in adult males doesn't fit with the general issue of ontogenetic diet shifts. | Yes | Revised to focus on the unique nature of this study and the rare occurrence of a large dataset for these species compared to other cetaceans (given they have fewer teeth, etc.). |
| Reviewer 2 | Sample size. In the introduction (line 105), 151 teeth are noted (presumably from 151 individuals?), while in the methods (line 126 and 130), 50 is noted (presumably from 50 individuals?), as on line 144 ('only 50'). Later (line 184) this 50 is reduced to a sub-set of 39, although it is unclear why the 50 with GLGs1-5 were reduced to those with GLGs1-3? Either way, would be good to bring up the original sample (151 individuals) examined and walk through the steps getting to the final sample. Similarly, the discussion (line 302) notes "across 50 individuals", but based on Figure 3 it seems this should be 39? In Figure 4 the sample size values for males and females should be explained in the caption, as these could easily be construed as the number of individuals (which presumably they are not). | Yes | See response to editor above |
| Reviewer 2 | Table 2. Good to sort this based on increasing AIC values. | Yes | As per response to editor above |
| Reviewer 2 | Table 3. Why is the 0.005 value in italics? For those values listed as 0.000 better to list as <0.001. | Yes | As per response to editor above |
| Reviewer 2 | Line 287-288 and Table 4. Good to be consistent with the number of decimal places shown for these values. | Yes | As per response to editor above |
| Reviewer 2 | Lines 113, 320, 333, 337, 367, 404. I'm not sure what the formatting requirements are for PLoS ONE but the use of common and scientific names is inconsistent (sometimes both, sometimes only one or the other), and would be good to be consistent. | Yes | As per response to editor above |
| Reviewer 2 | Line 470. This could be broadened to include population recovery in response to other sources of decline (e.g., MFAS, disease outbreaks). | Yes | As per response to editor above |
